# Texas Anti-Tuberculosis Association

**Published:** 1908-11

**Authors:** L. B. Bibb

**Affiliations:** Secretary, Austin, Texas


### Communications.

### Texas Anti-Tuberculosis Association.

COMMUNICATED BY L. B. BIBB, M. D., SECRETARY, AUSTIN, TEXAS.

The recent International Congress on Tuberculosis has greatly stimulated the public interest in the consumption problem. Texas participated liberally in the Congress. There seemed to be more delegates from Texas in Washington during the session than from any other distant State, and, owing to their neat and tasty badges, the Texans were conspicuous.

The Texas delegation numbered about a hundred. They started from Texarkana on a special train. The fact that all these people were interested in the subject enough to travel a thousand miles made it apparent that some permanent organization should be effected, and hence on the train en route to Washington the company of men and women banded together as the "Texas Anti-Tuberculosis Association."

Although it is a health organization, it is not meant to be composed of doctors and health officers alone. These latter will naturally gravitate into the organization; but there are not enough doctors and health officers to successfully combat this gigantic evil. Before we ever free ourselves of consumption, every man, woman and child must know how the disease is spread, and how it may be prevented. The doctors will look after the cure of cases already developed, but the public absolutely must be educated up to the point where it can help itself prevent the disease. How is it to be educated? By lectures, from the platform and from the phonographs, by traveling exhibits, by pamphlets, pictures, demonstration, but probably as much as anything by simple conversation. The everyday citizen must be impressed with a few simple facts about the necessity for controlling this disease, and the method of procedure. Then they will talk. Let women participate, too, as they seem to grasp more easily the fact that tuberculosis threatens every home. Then when the people get started to talking about tuberculosis and get interested in it, there is nothing that will multiply that interest so much as an organization in which every man and woman who desire may participate. Such was the association formed by the Texas delegation on September 26, 1908.

It was voted that all the sixteen hundred delegates appointed to the Congress, whether in attendance or not, should upon the payment of $1.00 membership fee be allowed the privilege of becoming charter members of the Texas Anti-Tuberculosis Association. There are no annual dues.

In selecting the officers of the association, it was thought best to place at the head of the organization a man whose occupation brings him in touch with a great number of people all over the State. With this in view, Mr. J. W. Graves, of Austin, president of a well known organization of traveling salesmen, was chosen. Mr. Graves possesses the additional qualification, that he is deeply interested in this question of consumption, and possibly no one in the State assisted the Texas committees more than he did in arousing the citizens to the importance of sending a good large delegation to the Congress.

Following the example of the Eastern and Northern States, the association will endeavor to provide and carry over the State one or more exhibits to set forth the various points concerning tuberculosis that need emphasizing. It will attempt to distribute pamphlets where they are needed, and to introduce more instruction into the schools along the line of prophylaxis in consumption. The association is affiliated with the National Association, with similar aims, and will encourage the organization of local societies at different points in the State. As soon as possible the association will attempt to bring about some amelioration in the condition of our consumptive poor throughout the State. It may do this through the agency of the various local organizations which, it is hoped, will soon be organized. The association will encourage legislation looking to the establishment of a State Tuberculosis Sanitarium for the poor, and possibly may advocate the enactment of a law requiring compulsory registration of all cases of tuberculosis occurring in the State. Its purposes are not entirely crystallized as yet, but will take such form as the exigencies demand. Meantime remittances for membership are coming in at the rate of forty or fifty a day.

The officers chosen to perfect the organization and conduct the affairs of the association are, with some minor changes, as follows:

OFFICERS TEXAS ANTI-TUBERCULOSIS ASSOCIATION.

*President.*

J. W. Graves, Austin.

*Vice Presidents.*

Mrs. Jos. B. Dibrell, Seguin.
Dr. W. S. Carter, Galveston.
Dr. Jno. S. Lankford, San Antonio.
Miss Katie Daffan, Dallas.
Dr. H. W. Cummings, Hearne.
Mr. George Sexton, Houston.
Dr. F. E. Daniel, Austin.
Mr. H. G. Wagner, Mineral Wells.
Dr. Frank Paschal, San Antonio.
Father J. M. Kirwin, Galveston.
Dr. M. M. Smith, Dallas.
Mr. W. B. Gilbert, Waco.
Mrs. W. A. Galloway, Dallas.
Dr. Ira C. Chase, Fort Worth.
Dr. Frank B. King, Houston.
Dr. Francis W. Gallagher, El Paso.

*Directors.*

Dr. F. U. Painter, Ch'm, Pilot Pt.
Dr. E. M. Thomas, Georgetown.
Dr. F. M. Hicks, San Antonio.
Dr. J. W. Greenwood, Memphis..
Mrs. J. S. Rowell, Pearsall.
Dr. T. J. Turpin, Corpus Christi.
Dr. J. J. Dial, Sulphur Springs.
Mrs. J. A. Stockton, Bartlett.
Dr. T. B. Fisher, Dallas.
Dr. S. A. Foote, Bay City.
Dr. W. C. Bryant, McKinney.
Dr. J. M. Loving, Austin.
Dr. W. M. Brumby, Austin.

*Executive Council, Board of Directors.*

Dr. W. M. Brumby, Ch'm, Austin.
Dr. J. M. Loving, Austin.
Dr. E. M. Thomas, Georgetown.
Dr. J. W. Greenwood, Memphis.
Dr. F. U. Painter, Pilot Point.
Dr. L. B. Bibb (Ex-Officio Secretary).

*Treasurer.*

T. C. Yantis, Brownwood.

*Secretary.*

Dr. L. B. Bibb, Austin.

*Committee on By-Laws.*

Dr. J. G. Pope, Chairman, Coleman.
Dr. Solon Milton, Fort Worth.
Mrs. Sam R. Scott, Waco.
Mrs. V. E. McFarland, Eagle Pass.
Dr. W. C. Hale, Jr., Dallas.
Dr. G. H. Moody, San Antonio.
Dr. S. C. Red, Houston.
Dr. B. F. Calhoun, Beaumont.
Mrs. H. D. Barnes, Tulia.

*Committee on Plans of Work.*

Dr. W. B. Russ, Chairman.
Dr. W. T. Jones, Fort Davis.
Dr. A. J. Beall, San Marcos.
Dr. D. Monroe, Cameron.
Dr. B. W. D. Hill, Dawson.
Dr. C. E. Davis, Linden.
Dr. R. R. Shappard, Anson.
Dr. F. D. Garrett, Gainesville.
Hon. H. H. Stephens, Texas.

*Committee on Legislation and Municipal Regulation.*

Dr. David R. Fly, Chairman.
Dr. J. G. Springer, San Antonio.
Dr. C. P. Yeager, Corpus Christi.
Dr. L. Coons, Wichita Falls.
Dr. H. S. Rhu, San Angelo.
Mr. H. R. Casparis, Austin.
Dr. W. T. Reeve, Boerne.
Dr. W. R. Blalock, Dallas.
Hon. Morris Sheppard, Texarkana.

*Committee on Education and Publicity.*

Dr. M. M. Carrick, Chairman.
Dr. Theo. Y. Hull, San Antonio.
Dr. W. H. Anderson, El Paso.
Dr. L. A. Grizzard, Abilene.
Mrs. R. R. Shappard, Anson.
Dr. N. J. Phenix, Colorado.
Miss Maude Casparis, Austin.
Dr. G. W. Baskett, Van Alstyne.
Mrs. C. F. Collins, Clifton.

*Committee on Finance.*

Miss Elizabeth Holloway, Chairman.
Mrs. Emma B. Shindler, Dalhart.
Dr. C. L. Johnson, Dallas.
Dr. S. Burg, San Antonio.
Dr. R. May, Whitewright.
Dr. J. H. Taylor, Marshall.
Dr. O. F. Gober, Temple.
Dr. J. P. Gibbs, Houston.
Mrs. Thekla M. Brumby, Austin.

*Committee on Resolutions.*

Dr. W. L. Crosthwait, Chairman.
Dr. C. L. McClelland, Rockport.
Dr. W. D. Patton, Amarillo.
Dr. H. D. Barnes, Tulia.
Mrs. W. C. Hale, Jr., Dallas.
Dr. J. M. McDuff, Atlanta.
Dr. N. H. Ellis, Midland.
Dr. F. L. Barnes, Trinity.
Miss Ada Olivia, Houston.

### Abstracts and Selections.

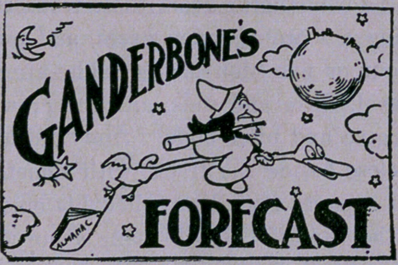

**For November.**

(Copyright 1908, by C. H. Rieth.)

In the old Roman calendar November was the ninth month. Blessings fell early, and the empire gave thanks just before the first frost; but about 700 B. C. the trusts left the people so little to be thankful for after nine months that it was decided to wait a while and see if anything would come of the Roman elections. Numa accordingly made November the eleventh month and had Thanksgiving fall with the first snows, notwithstanding the month gets its name from the Latin novem (nine).

The frisky colt will sniff the air and hear the whistling quail, and the festive calf will indicate the zenith with his tail. The frost will paint the forest with a deep and redder dye, the hired man will shuck the corn, the pumpkin vine will pie, the politicians will hit up their office-holding feud, and the modest maple tree will blush and come out in the nude.

